# Polymeric Microspheres for Medical Applications

**DOI:** 10.3390/ma3063537

**Published:** 2010-06-07

**Authors:** Ketie Saralidze, Leo H. Koole, Menno L.W. Knetsch

**Affiliations:** Department of Biomaterials and Biomedical Engineering, Maastricht University, P.O. Box 616, 6200 MD Maastricht, The Netherlands; E-Mails: l.koole@bioch.unimaas.nl (L.K.); menno.knetsch@bioch.unimaas.nl (M.K.)

**Keywords:** microspheres, polymer, bulking agent, embolization, drug-delivery

## Abstract

Synthetic polymeric microspheres find application in a wide range of medical applications. Among other applications, microspheres are being used as bulking agents, embolic- or drug-delivery particles. The exact composition of the spheres varies with the application and therefore a large array of materials has been used to produce microspheres. In this review, the relation between microsphere synthesis and application is discussed for a number of microspheres that are used for different treatment strategies.

## 1. Introduction

The number of medical applications for synthetic biomaterials continues to expand. One particularly interesting development concerns the use of injectable polymeric biomaterials. The forms in which the biomaterials are injected can vary from particulate matter, e.g., microspheres or irregularly shaped particles, to gels and cements that are injected in liquid form and which harden inside the body. There are many different applications of injectable biomaterials in a number of minimally invasive approaches. These can be generally divided into four different types of treatments, namely:

1) fillers for soft tissue, e.g., to correct wrinkles and lips, to treat lipoatrophy in AIDS patients, to treat acne scars, or fillers for hard tissues, as would be used in vertebroplasty, the stabilization and restoration of collapsed/fractured vertebrae by injection of a cement [1,2,3,4,5].
2) bulking agents in soft tissues to augment the efficiency of opening-closing systems, e.g., for treatment of stress urinary incontinence (SUI), vesicoureteral reflux, or for vocal cord augmentation. The injected biomaterial enhances tissue stiffness and gives support to the muscles responsible for opening and closing tubular structures [3,6,7,8].
3) particles for embolization therapies that purposefully occlude blood vessels. Embolization therapy can be applied to combat the growth and development of solid tumors by blocking the feeding artery. Occlusion of blood vessels can also be required in case of arterio-venous malformations or hemoptysis (severe bleeding) [9,10,11].
4) as drug-delivery depots for a strictly controlled drug delivery. These can be gels that form inside the patients, or particles that slowly degrade after injection while releasing the drug during their erosion [12,13,14,15,16,17].

Moreover, combinations of different applications are being used today. An example is trans-arterial chemical embolization (TACE), an application in which particles are injected into the feeding artery of a tumor. The particles are loaded with an anti-cancer drug so that they will a) block the blood supply to the tumor and b) release cytotoxic drugs in high concentrations locally inside the tumor [18,19]. Another example would be the injection of microspheres, as a bulking agent, which are covered with collagen and growth factors, to induce tissue formation and regeneration, while enhancing the efficiency of the therapy and diminishing the risk of particle migration [20].

The ultimate injectable biomaterial would be in the form of a material that *in situ* will form a scaffold for cells to grow on. The desire is for these cells to differentiate into types that possess a particular function, so as to restore disturbed or lost organ function. For this, the material should combine mechanical characteristics with biofunctionality on the surface, and possibly with degradation characteristics.

It is clear that the increased use of injectable biomaterials has coincided with developments in imaging and minimally invasive treatments. The exact fate of the biomaterials is of critical importance for the efficiency and safety of the treatment. X-ray and magnetic resonance equipment with increased sensitivity, as well as spatial and temporal resolution, make it possible to follow the fate of injected biomaterials during and after injection into the intended site of the patient. Consequently, the requirements for the biomaterials that are used in minimally invasive treatments have changed and have become more stringent. The clinical use of polymeric microspheres as injectable biomaterials has increased steadily over the past few decades. Polymeric microspheres have gained in popularity because synthetic microspheres can be easily produced with well-defined physical parameters and in each desired size range. Additionally, the straightforward control over dimensions of the injected particles makes them predictable and easier to inject. Also, microspheres pose a great opportunity to be used as reservoirs for drugs and carriers of bioactive molecules on their surface.

In this review we will focus on the synthesis of polymeric microspheres and how these are currently applied clinically. We will discuss the use of synthetic microspheres for two different types of treatment, namely bulking agents and embolization therapy. Furthermore, we will describe how localized drug delivery from microspheres may improve therapeutic efficiency. In this review we aim to describe how careful design of synthetic polymeric microspheres can lead to novel, more efficient and safer treatment protocols in the clinic.

## 2. Microsphere Synthesis

Microspheres are defined as spherical microscopic particles that range in size from 1–1,000 µm [16,21]. The definition on the basis of size can sometimes be confusing since spheres with a size of over 1,000 μm are still often called microspheres. These particles have a wide variety of possible applications, that range from use in the medical field, application as carrier materials for purification purposes in the biochemical sciences [22], to use as flow indicators [23,24]. Non-polymeric microspheres are mainly produced in a simple method based upon the formation of spheres in an aqueous environment and subsequent drying, and if required, sintering [25,26].

**Figure 1:**
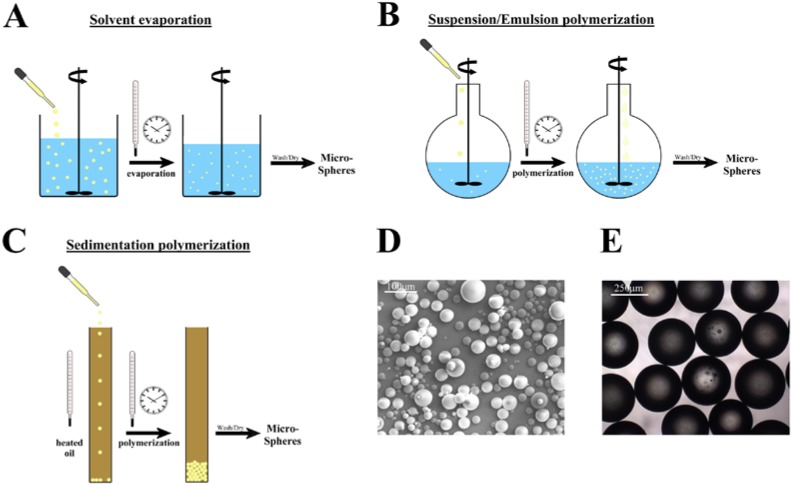
Schemes of different synthetic polymeric microsphere synthesis routes. Details of the different methods are given in the text. A) solvent evaporation; a polymer solution is dripped into an immiscible phase and after evaporation of the solvent, microspheres remain. B) Suspension/emulsion polymerization; A mixture of monomers is dripped into a stabilizing solution and polymerization is induced to form regularly shaped polymer microspheres. The composition of the stabilizing liquid and the added solution are shown in Table 1. C) Sedimentation polymerization; a monomer solution with initiator are carefully added to a heated oil column, and the resulting microspheres end up at the bottom of the column, where polymerization is allowed to be completed. D) A scanning electron micrograph of polymeric microspheres produced by solvent evaporation and E) Phase contrast micrograph of microspheres synthesized by suspension polymerization.

Here we will focus on medical microspheres that are composed of polymers. The use of polymers for synthesizing microspheres enables the production of uniformly shaped and well-defined spheres in a wide range of sizes. Polymeric microspheres can be synthesized using a variety of different methods, some of which are depicted in Figure 1. Microsphere synthesis can be roughly divided into two different strategies. The spheres can be produced from linear pre-existing polymer chains or can be formed during the polymerization process, starting from a monomer solution [16].

### 2.1. Microspheres from linear polymers

Methods using linear polymers as starting materials are especially well suited for preparing microspheres from polymers that are not formed via radical polymerization, like poly(lactic acid), poly(glycolic acid), or poly(ε-caprolactone) [15,27]. Also, naturally occurring polymers can be formed to microspheres by these methods. The most commonly used technique is solvent evaporation [16,28]. For this, the polymer is dissolved in a more or less volatile solvent, and carefully dripped into a solution with a non-miscible fluid, the continuous phase. This phase contains a stabilizer to ensure the formation and maintenance of a spherical shape (Figure 1A). The solvent will slowly evaporate, and as a result, solid polymeric microspheres will remain dispersed. These spheres can be sedimented and washed free of stabilizer. An example of such microspheres produced by solvent evaporation is given in Figure 1D [29].

Another example is the formation protein microspheres, for instance, consisting of fibrin as described by Gorodetsky [30,31]. To obtain fibrin microspheres, the precursor protein fibrinogen is dissolved in water and the enzyme thrombin is added. Thrombin is a serine protease that cleaves fibrinogen and causes polymerization of the proteolytic products, leading to formation of a stable fibrin polymer mesh. The solution of fibrinogen and thrombin is dripped into stirred, heated (corn) oil, and the enzymatic reaction is allowed to proceed. After the water has evaporated, the solid fibrin microspheres can be collected and washed.

Naturally occurring polymers like cellulose, chitosan, and proteins such as collagen and albumin, can also be used to produce microspheres [32,33,34,35,36]. For this, the polymer or protein is dissolved in an aqueous medium and dripped into an oil phase under continuous stirring or sonication. Mixing of the two phases results in the formation of water-polymer droplets in the oil. The intermolecular forces then stabilize the microspheres, or denaturation will occur in the aggregate, rendering the polymers insoluble in the water phase. Alternatively, the addition of cross-linking agents will link the polymers together and ensure the mechanical stability of the microspheres. Here we will not discuss these polymers in detail since we want to concentrate on microspheres from synthetic biomaterials.

The major advantage of solvent evaporation synthesis of microspheres is that it is relatively simple; virtually all dissolved polymers can be used and high yields can be obtained. The disadvantage is that control over sphere size between different batches is complicated. Also, the used solvent and surfactant are often toxic and need to be removed before the spheres can be applied *in vivo*. These washing steps are time-consuming and result in a large volume of waste. Furthermore, drug-loaded spheres may lose some of the loaded drug during such washing steps [15,28].

An alternative to these liquid in liquid solvent evaporations is the spray drying technique [37,38] The polymer is dissolved in a volatile solvent, and under pressure, a fine spray is generated, which is dried under high power heating, forming loose spherical particles. The technique is easy and straightforward to upscale. A disadvantage of this technique is that the resulting microspheres display high polydispersity. Additionally, relatively high temperatures are required for the drying, and therefore, more sensitive bioactive compounds can be severely damaged during the spray-drying process [37,38,39].

### 2.2. Microsphere synthesis from radical polymerization

Alternative methods of producing microspheres are based upon a mixture of monomers and polymerization initiator that are formed into spheroids in a stabilizing non-miscible phase. Several different methods have been developed and the most common of these will be discussed here: suspension, emulsion, dispersion, and sedimentation polymerization [16]. For suspension polymerization, monomers are mixed with an initiator and dripped into a non-miscible phase, most commonly water, with surfactants or detergents as stabilizers, to ensure formation of proper spheroid particles [16,40] (Figure 1b; Table 1). The solution is heated to induce activation of the initiator and the start of the radical polymerization reaction. After the polymerization reaction has terminated, microspheres can be collected and washed free from the stabilizers. The technique is rather simple, and most monomer mixtures can be used. Also, monomers that are partly soluble in water can be used, although a more complex stabilizer solution is required in such cases [41]. With this technique, a wide range of microsphere sizes can be obtained, roughly ranging from 40–1,000 µm.

The major disadvantage of suspension polymerization is that the resulting microspheres display relatively high polydispersity [16]. Micro-sieving of the spheres is then required to obtain spheres in well-defined ranges. This leads to reduced yields, although in practice, there are applications for a wide range of sizes.

An alternative method is emulsion polymerization (Figure 1b; Table 1) [16,21,42]. For this technique, the initiator is dissolved in the solution that contains emulsifiers and stabilizing molecules. These are necessary to obtain an emulsion of monomers in the medium, which means that micelles with monomers are formed. The monomer is also present in the solution as droplets and dissolved (in a very low concentration). Since the initiator is only present in the water phase, the initiation of polymerization will involve the small number of dissolved monomer molecules. The formed free oligomeric radicals will then be adsorbed by the monomer-containing micelles, or become surrounded by dissolved monomer and surfactant molecules. In this way, micelle particles become centers of polymerization that will be fed by monomers from the droplets in the medium. The reaction is terminated when all the monomers are consumed. Subsequently, the surfactant is washed away, leaving clean microspheres. This emulsion polymerization is especially useful for the synthesis of small particles, nano-spheres, and microspheres (range 0.1–10 µm). The obtained microspheres display low polydispersity and high yields can be obtained.

Another method of microsphere synthesis is dispersion polymerization [16,21,43]. Some groups also refer to this technique as phase-separation polymerization [44]. For this, a solution of the monomers and initiator, together with a stabilizing molecule, in a single, miscible phase, is prepared. The solvents are chosen such that the resulting polymer is insoluble in the solvent. In this way, the polymerization can proceed in small droplets that are stabilized by the stabilizing molecule, which also prevents the aggregation of the polymerizing particles. The soluble monomers are gradually converted into an insoluble polymer. The exact composition of the solvent mixture and the speed of stirring and heating are all important parameters for the resulting sphere size [44]. Thus, careful fine-tuning of these parameters is required to obtain microspheres with a defined size. This method is well suited for relatively small microspheres (range 1-10 µm).

**Table 1:** Composition of the stabilizing phase and the monomer-solution for suspension polymerization. The monomers are mixed with the stirred, stabilizing solution for the synthesis of polymeric microspheres.

	Stirred solution	Added solution
Suspension polymerization	Surfactant	Monomers
		Initiator
Emulsion polymerization	Surfactant	Monomers
	Initiator	
Dispersion polymerization	Surfactant	-
	Initiator	
	Monomers	

There is a technique available to obtain large size microspheres (400–2,000 µm) with low polydispersity, namely sedimentation polymerization (Figure 1c) [45,46] The monomers are mixed with an initiator and exactly measured drops are dripped into a column of heated mineral oil. The droplets will slowly sediment, and during the sedimentation, the polymerization will proceed and ensure that the microspheres, which gather at the bottom of the column, will not stick together. They are already partly polymerized, especially on the outside, where the temperature was initially highest, and are therefore not sticky. The ratio of the monomers and the initiator is of critical importance for the integrity of the microspheres during the procedure. The gelation and hardening of the microspheres has to be fast enough so that by the time the droplets hit the bottom, the microspheres are hard enough to keep their shape. The size of the needle and the speed with which the monomers are dripped into the heated oil column, determine the size of the monomer droplets and the resulting microspheres (400–2,000 µm) [45,46]. The technique is not widely used, but may be a proper alternative when large size microspheres with low polydispersity have to be synthesized. Of course it is difficult to envisage a large scale production of such microspheres using sedimentation polymerization since slow addition to the column of oil is essential to ensure that the formed microspheres stay separate and do not form aggregates at the bottom of the column.

## 3. Clinical Applications of Microspheres

The use of microspheres as injectable biomaterials has become more and more popular over the last few decades. The tight control over the shape and dimension of injected particles makes them ideally suited for treatments in which the particle size is of critical importance. Also, non-spherical particles suspended in a carrier fluid or paste, have a tendency to aggregate, making injection difficult and impractical [2]. Microspheres have a controlled shape and size and behave very predictably during the injection procedure. Of course, microspheres need to possess a number of characteristics that are required for clinical, *in vivo* use. The microspheres should be biocompatible, safe, stable, display desired functionality inside the patient, and should demonstrate desired and predictable degradation kinetics [47,48,49]. All these parameters are determined by the physico-chemical nature of the synthetic microspheres. Also the chemical modification of the surface of the microspheres can be of critical importance for optimal functionality *in vivo* and for the induction of the desired response within the surrounding tissue. When the microspheres are designed to demonstrate controlled drug release, the exact kinetics of the release should be determined and fine-tuned, which can be achieved by careful design of the microsphere composition. In some cases the *in vivo* degradation of the microspheres is desired and the local effect of the degradation products should be considered as well, to avoid severe complications after application of such biodegradable spheres.

As stated earlier, clinical use of synthetic microspheres can be roughly subdivided into several applications: 1) fillers and bulking agents, 2) embolic particles, and 3) drug delivery vehicles. Here we will discuss each of these applications for which the use of microspheres as an alternative, minimally-invasive treatment has been studied extensively, and in the use of which in clinical practice has occurred for a number of years.

### 3.1. Microspheres as fillers and bulking agents

Fillers and bulking agents aim to replace tissue volume that has been lost because of disease, injury, or more commonly, aging. The interventions that make use of fillers and bulking agents range from the augmentation of lips and treatment of wrinkles in cosmetic surgery, to the treatment of lipoatrophy in HIV-patients, *i.e.*, the loss of facial subcutaneous fat in plastic surgery and stress-urinary-incontinence (SUI) in urology [1,2,3,4]. This means that there is a wide range of possible applications; thus, the characteristics of the injected biomaterials (here we discuss microspheres) varies. The injected fillers and bulking agents can be divided in several categories depending upon the desired behavior of the biomaterial after injection in the patient [1,50]. The first category aims at replacing volume lost by aging of tissue and at minimal response of the surrounding tissue. Also, the material should be absorbed and degraded by the patient within a certain time-span. This is to avoid long-term complications with granuloma formation, which can disturb the effect by formation of hard and fibrous tissue around the implanted materials [50,51,52]. Bluntly said, these microspheres aim to merely take up space. The injection has to be repeated after certain times to ensure continuity of the therapeutic or cosmetic effect. Another group of materials aims at stimulating the growth and repair of tissue. The injected microspheres then also have to be degradable, but at the same time, actively interact with the surrounding tissue to enhance cell growth, so that newly formed cells will occupy the volume that was originally lost, and which is now taken up by the injected microspheres. The last category encompasses the permanent fillers and bulking agents. These include synthetic, polymeric microspheres that are non-degradable, and which stay at the site of injection for the remainder of the patient’s lifetime. The characteristics of such permanent microspheres are of course very different from those of the other two categories. These materials are non-degradable and have to show mechanical properties that are adapted to the intended application. They have to display the desired softness and strength so that optimal functionality is ensured. For instance, bulking agents that aim at restoring open-closing systems improving the stiffness of tissue surrounding closure muscles have specific requirements. Microspheres that are too soft will not result in satisfactory closing, while spheres that are too hard or too big may feel uncomfortable for the patient. Also, migration away from the injection site should be avoided, since such migration can lead to serious complications like pulmonary embolism or even stroke [52,53,54]. It is therefore of importance that the surgeon can detect the microspheres during and after the injection procedure. This enables him/her to avoid misplacement of the spheres. In addition, the injection of the right amount can be monitored more accurately, along with the result after injection.

This means that the microspheres have to be intrinsically radiopaque, *i.e.,* X-ray visible, or that they have to be suspended in a carrier that is radiopaque. There have been reports on microspheres that are intrinsically radiopaque.

**Figure 2:**
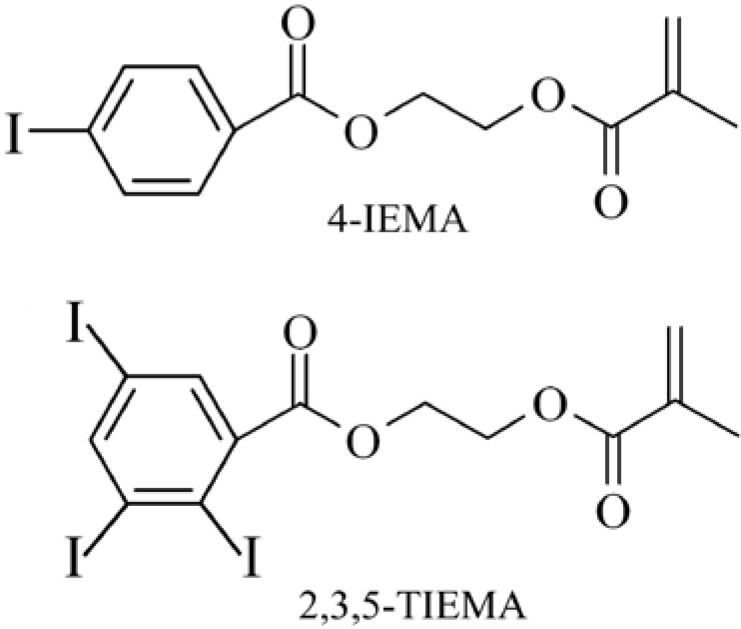
Chemical structure of the iodine containing methacrylic monomers 2-[4’-iodobenzoyloxy]ethyl methacrylate (top) and 2-[2’,3’,5’-triiodobenzoyloxy]ethyl methacrylate (bottom). These monomers render polymers radiopaque.

This has been achieved by incorporation of iodine containing monomers 2-[4’-iodobenzoyloxy]ethyl methacrylate (4IEMA) or 2-[2’,3’,5’-triiodobenzoyloxy]ethyl methacrylate (TIEMA) into randomly polymerized microspheres (Figure 2) [29,55,56].

Finally, to induce the desired response of the surrounding tissue, it may be necessary to modify the surface of the microspheres with bioactive compounds. This means that the exact composition of the microspheres has to be fine-tuned to suit the application as well as possible. Here we will discuss the use of fillers and bulking agents by the example of stress-urinary-incontinence (SUI).

#### 3.1.1. Microspheres in minimally-invasive treatment of stress-urinary-incontinence (SUI)

**Figure 3:**
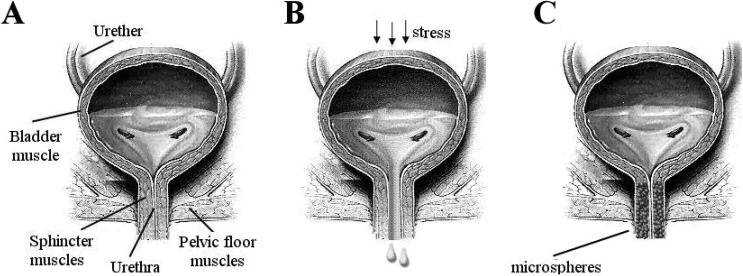
Front view of bladder. a) Strong sphincter and pelvic muscles keep the urethra closed. b) Weak muscles result in urine leakage. c) Complete coaptation of the urethra, which is achieved by injection of the bulking agent around the urethra.

Stress urinary incontinence is defined as follows:

Stress urinary incontinence (SUI) is the complaint of involuntary leakage on effort or exertion, or sneezing or coughing [57]. Incontinence has been known as long as written records were being made. Already in the 2nd millennium BC, the Egyptians described a series of recipes and treatments for incontinence [58,59]. Nowadays, more than 200 million people worldwide live with incontinence. A major subset of these patients, approximately 35%, suffers from so-called stress urinary incontinence. SUI is a bladder storage problem in which the strength of the muscles (urethral sphincter) that help to control urination is reduced (Figure 3).

The sphincter is not able to prevent urine flow when there is increased pressure from the abdomen. The disease is usually caused by weakening of the pelvic floor muscles that support the bladder and urethra or by the malfunction of the urethral sphincter (intrinsic sphincter deficiency). The weakness may be caused by prior injury to the urethral area, child birth, neurological injury, medications, or surgery.

#### 3.1.2. Treatment of SUI

In 1948 Arnold Kegel introduced pelvic floor muscle training (PFMT) for management of SUI [60]. The aim of these exercises, which may actually be rooted in Chinese Taoism, is to strengthen the muscles that support the urethra, the bladder sphincter muscles, via the permanent elevation of the levator plate into a higher location inside the pelvis. This increases muscle volume, strengthens connective tissue in the muscles, strengthens bony connections, and leads to the more effective recruiting of motor neurons [61]. Long-term results of the PFMT program are unclear. Symptoms tend to worsen on the long run and women then prefer alternative treatments [62]. Another considerable argument against this method is the conviction that PFMT must be done permanently. But because of the lack of risk and relatively small cost, PFMT is recommended by health professionals as a first-line therapy [63].

Pharmacological treatment of SUI has been attempted, but with moderate success. The mixed serotonin/noradrenaline reuptake inhibitor Duloxetine was used. Duloxetine was believed to increase the strength of urethral sphincter contraction and to increase bladder outlet resistance, thereby preventing accidental urine leakage. However, Duloxetine has a wide spectrum of severe side effects, which led to denial of approval by the US Food and Drug administration [64,65,66,67,68,69].

There are also surgical interventions, which have been practiced for decades. In 1996, Ulmstein first introduced the tension-free vaginal tape (TVT), which is used as a sling [70]. This procedure is performed through a small vaginal and two small abdominal incisions. TVT involves placing a narrow strip of synthetic material around the middle of the urethra. Surgical intervention of SUI has been associated with some serious complications like perforation of the bladder [71], excessive bleeding [72], erosion of the sling into the vagina or urinary tract [73,74,75], and infection. Urethral erosion is dependent upon the biomechanical and mesh properties of the tape, as well as on surgical technique. Complications result in pain and worsening of SUI symptoms [76,77,78,79]. When the implanted biomaterial meshes extrude from the tissue, infection is almost unavoidable and the sling has to be removed. Furthermore, bone anchors used in SUI surgery can be associated with pubic osteomyelitis and osteitis pubis. In such cases, surgical removal and aggressive treatment with long-term antibiotics is required [80,81].

#### 3.1.3. Modern minimally invasive therapies for SUI

During the last two decades, less invasive procedures, aimed at achieving high long-term cure rates for SUI, have been developed. Most successful are peri-urethral injections of a so-called bulking agent. This technique led to very good results, especially for the elderly patients, for women who have undergone multiple failed procedures, or after radiotherapy, where the urethra may have become fixed and scarred.

From the perspective of biomaterials science, these developments are particularly interesting. A variety of different bulking agents have been used; the most commonly used materials are cross-linked bovine collagen (Contigen), polydimethylsiloxane (PDMS, Macroplastique), ethylene vinyl alcohol copolymer (Tegress), polytetrafluoroethylene (PTFE, Urethrin), carbon-coated zirconiumdioxide beads (Durasphere), calcium hydroxyapatite (Coaptite), and dextranomer/hyaluronic acid copolymer. Bulking agents are injected carefully in the periurethral tissue. The goal is urethral coaptation during the storage of urine, maintenance of that coaptation during periods of increased abdominal pressure, and the improvement of sphincter closure. Degradable bulking agents such as autologous fat tend to relieve symptoms, but have comparatively low efficiency and disappointing long-term results. In addition, some side-effects were reported, such as granuloma formation, obstructive mass formation, urine retention, and fat embolism [82,83,84,85,86,87,88,89].

Ethylene vinyl alcohol copolymer suspended in DMSO (Tegress) was evaluated as an embolic agent, but was also approved as a bulking agent for treatment of SUI. Recent studies not only demonstrated that Tegress may be less efficacious than reported in FDA trials, but also that a significant percentage of patients experienced serious complications like urethral erosion [90,91]. Injection of solid polydimethylsiloxane (silicone rubber) particles proved to have moderate success, with approximately half of the patients being cured after 24 months [92,93,94]. However, the misplacement or injection of too many silicon particles leads to complications, caused by the invisibility of the particles [95,96].

Polytetrafluoroethylene paste is a resin with very high molecular weight and high viscosity. The material is composed of small particles and has been used to treat SUI since 1964. In spite of this, PTFE is not approved in the United States for treatment of incontinence, because of dangerous complications, such as distant migration, periurethral abscess, urethral diverticulum, urethral granuloma formation, and even increased tumor risk [97,98,99,100].

Durasphere^®^ is composed pyrolitic carbon-coated zirconium oxide beads suspended in water–based carrier gel containing beta-glucan. Pyrolytic carbon is inert and biocompatible, and is used in implantable medical devices, including replacement heart valves [101]. One of the potential advantages is its very low immunogenicity. Durasphere^®^ particles are relatively large, 200–500 μm, and radiopaque, which is useful for tracing after injection. Durasphere^®^ is non-degradable, therefore concerns were raised over long-term durability [102]. In spite of promising clinical data, Durasphere^®^ demonstrated some serious complications, among which migration from implantation site is the most dangerous one. The obvious dislocation from the implantation site was shown in animal studies [103]. Six months after injection of carbon coated beads with diameter of 251 to 300 μm, significant migration of these beads was observed into local and distant lymph nodes, as well as into the urethral mucosa. The exact reason for bead migration remains unclear [104]. Durasphere^®^ injection may result in long-term outlet obstruction, which can cause voiding dysfunction, permanent urinary retention and periurethral mass formation [105,106,107]. Urethral prolapse is an uncommon condition, but some cases have been described [108]. The long-term efficacy of Durasphere^®^ injection is low. At 18 months follow up in 70 patients only 13% of patients considered themselves cured, 52.2% improved and 34.7% failed [109]. Another study demonstrated that Durasphere^®^ remained effective in 35%, 33%, and 21% of patients at 12, 24, and 36 months respectively [110].

Synthetic calcium hydroxylapatite (CaHa) spheres suspended in a water-based gel carrier is a sterile, radiopaque, non-pyrogenic, semi-solid, cohesive implant. It is also known as Coaptite and Radiesse. Coaptite is used for treatment of SUI and Radiasse is used for the correction of facial lypoatrophy in HIV patients [111,112]. They differ in size range of the microspheres.

Theoretically, injected CaHa spheres have to form a scaffold for developing soft tissue that will gradually replace the gel carrier. For integration in the surrounding tissue, fibroblasts have to attach to and grow on the surface of the spheres, resulting in anchoring at the injection site. Coaptite had promising results beforehand [113]. Nevertheless, the implant also has shortcomings. The histology of injected CaHa microspheres demonstrated that microspheres become deformed, appearing irregular, and start being adsorbed at nine months, likely because of enzymatic breakdown of the calcium hydroxylapatite [114]. Furthermore, it looks as if CaHa microspheres migrate away from the injection site. This migration may result in periureteral fibrosis, ureteric obstruction, and subsequent renal loss [115]. Moreover, a granulomatous reaction can lead to urethral prolapse as early as 3 months after the transurethral injection of calcium hydroxylapatite [116].

### 3.2. Microspheres for embolization therapy

Embolization is defined as the “therapeutic introduction of various substances into the circulation to occlude vessels, either to arrest or prevent hemorrhaging, to devitalize a structure, tumor, or organ by occluding its blood supply, or to reduce blood flow to an arteriovenous malformation” [117].

**Figure 4:**
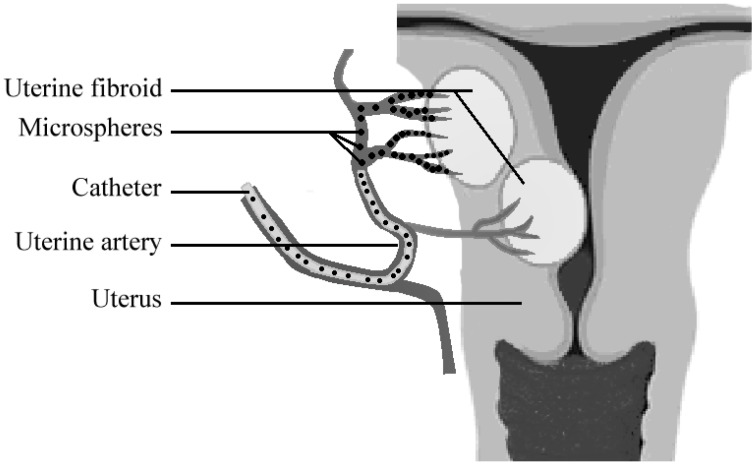
Scheme of uterine artery embolization (UAE). An angiographic catheter is introduced via the femoral artery, through a small opening in the groin. Microspheres are injected into the uterine artery that is supplying the fibroids. The microspheres subsequently block these arteries and induce shrinkage of the fibroid.

Closure of a target artery can be achieved by use of different embolic agents, such as synthetic microparticles, pellets, glues, or platinum coils [118,119]. This technique, also known as embolotherapy, leads to the obstruction of arterial blood flow. Injected particles act in the same way as thrombotic emboli (Figure 4) [120,121,122,123,124].

The field of interventional radiology routinely uses artificial microparticles and live-imaging techniques to specifically block arteries and to starve the target tissues of oxygen and nutrients. For this, a catheter is inserted and maneuvered to the target vessel that has to be embolized. Subsequently, the embolic particles are injected into the flowing blood and become stuck in the vessel, thus blocking the blood flow to the downstream tissue.

Embolotherapy is used in a variety of treatments of: (i) tumors, varicoceles, and organ ablation; (ii) hemorrhages, e.g., pelvic, posttraumatic, epistaxis, and hemoptysis; (iii) vascular anomalies, e.g., venous, lymphatic, and arteriovenous malformations. The treatment of inoperable tumors is also among the applications of embolotherapy. Tumors that lie deep in the brain are an obvious target for embolotherapy, although one has to keep in mind that such interventions are often more palliative. Furthermore, benign tumors like uterine fibroids can be treated by embolizing the feeding artery as an alternative to the common hysterectomy. Thus, in this case, embolotherapy is a minimally-invasive alternative to surgery. Another application of embolic particles is the treatment of haemoptysis, *i.e.*, severe bleeding because of trauma or of infections in the lungs, e.g., pneumonia can cause heavy bleeding. Embolization in such cases is used to stop lethal blood loss from the patient [119,125,126,127,128,129,130,131,132].

#### 3.2.1. Embolization of tumors

Embolotherapy is a new minimally invasive technique for the treatment of solid tumors. The embolization is performed pre-operatively to shrink the tumor and prevent excessive blood-loss, or as a palliative treatment of inoperable tumors. Benign uterine tumors in women, called uterine fibroids (UF), will be discussed to explain tumor embolization in further detail. The treatment of hepatocellular carcinoma (HCC) with drug-eluting microspheres will be discussed later in this review, because this treatment combines embolization with the controlled local delivery of drug to enhance the efficacy of the treatment.

UF consist mainly of smooth muscle and large amounts of extracellular matrix containing collagen, fibronectin, and proteoglycan. UF are also called fibromyomas, leiomyomas or myomas. They are very common and clinically apparent in about 25% of women, but because many commonly used imaging techniques lack resolution, the true prevalence may be as high as 77% [133].

UF can cause abnormal uterine bleeding, resulting in iron-deficiency anemia, dysmenorrheal, and non-cyclic pelvic pain [134,135,136,137]. The enlarging pelvic mass contributes to urinary problems and constipation [138,139,140]. UF are also associated with an increased risk of complications during pregnancy, and with infertility [141,142]. In other words, UF have a significant impact upon the quality of life of women [143].

Treatment of UF is relatively crude; surgical removal of the uterus, hysterectomy, is the most common method. In the US only, approximately 600,000 hysterectomies are performed annually, with uterine fibroids accounting for approximately 40% of all hysterectomies [144]. Especially for younger women, hysterectomy means that the possibility for child birth is gone and this may have serious psychological effects. Also, hysterectomy often does not relieve symptoms in several women, and some women reported new symptoms, such as hot flashes, weight gain, and depression [145,146].

Myomectomy is the surgical removal of uterine fibroids with preservation of the uterus. This is an invasive surgical technique, which can be performed in several different ways depending upon the size, number and location of the UF. There are three approaches to myomectomy: abdominal, laparoscopic, and hysteroscopic. Despite preserving fertility, myomectomy has complications. Most common complications of myomectomy are damage and infections of surrounding organs; damage, weakening and scaring of womb, which can lead to complications during the pregnancy such as rupturing the womb wall; and bleeding, which can lead to full hysterectomy [147,148,149].

Medications for uterine fibroids do not eliminate fibroids, but may shrink them. These medications target hormones that regulate the menstrual cycle, and treat symptoms such as heavy menstrual bleeding and pelvic pressure. The most commonly reported adverse effects of progesterone receptor modulators are headache, abdominal pain, nausea, dizziness, and metrorrhagia [150]. Side effects such as muscle cramps, acne, fluid retention, unwanted hair growth, weight gain, and a deeper voice can occur during androgen therapy [151].

Embolotherapy is therefore an important alternative for the treatment of uterine fibroids. This means that the feeding arteries of the fibroids are blocked by localized injection of microparticles (Figure 4).

As a consequence, the fibroids will become devoid of oxygen and nutrients. The mass of the UF will diminish over time and the symptoms will thus be reduced (Figure 4). The interventional radiologist has the choice of only a limited number of synthetic microparticles that can be used for the embolotherapy.

Embolotherapy was long performed with PVA particles that were irregular in shape, and therefore almost impossible to calibrate [9]. These particles had a strong tendency to form aggregates and also did not always lead to complete blockage of the targeted blood vessel. The introduction of synthetic polymeric microspheres to this field greatly enhanced the efficiency of embolization therapy. The embolic particles could now be well calibrated and the interventional radiologist now had the possibility of choosing, based upon the diameter, a size range of spheres with which to specifically block a vessel. Although it is clear that polymeric microspheres are of vital importance for embolization therapy, no new, sophisticated materials have been introduced in this field. Actually, PVA is still one of the favored materials [9,152]. During the last 15 years, these microspheres have found general use for targeted embolization. There are commercial microspheres available composed of trisacryl-gelatin (Embospheres, Biosphere Medical, Rockland, MA, USA), PVA (Contour SE; Boston Scientific, Natick, Ma, USA), and Bead Block (Biocompatibles, Farnham, UK) [153,154,155]. All these microspheres are hydrophilic. It is important for microspheres to have some elasticity, since the microspheres for embolization have to pass through a long (micro)-catheter. Hydrophobic microspheres tend to aggregate and stick to the wall of the catheter, making injection cumbersome and difficult to control, since the interventional radiologist has to use too much force to get the microspheres through the catheter [9]. The elastic spheres however, are easily pushed through the catheter and therefore a more controlled delivery of the spheres can be obtained.

However, these commercially available PVA or trisacryl-gelatin microspheres are not ideal because they are not visible under standard clinical conditions (X-ray). The lack of radiopacity, *i.e.*, X-ray visibility, forces the interventional radiologist to guess how much material has been injected and where the particles end up. Therefore, the currently used microparticles are mixed with liquid contrast agent to at least generate an idea of where the injected particle suspension ends up, although no information on the actual fate of the microspheres is available. As described earlier (part 3.1), intrinsically radiopaque microspheres, containing iodine-monomers 4-IEMA or TIEMA (Figure 2) are available [54,55]. The microspheres with intrinsic radiopacity are also available as hydrophilic spheres, but no data is available concerning the injectability and *in vivo* efficiency. Such microspheres with intrinsic radiopacity may help the interventional radiologist to avoid non-target embolization, which happens in a significant number of cases [156]. The fate of the injected microspheres can be monitored exactly during and after their injection, and the procedure can be stopped or interrupted if particles move in the wrong direction. This is a major improvement upon the currently available microspheres that are radiolucent, *i.e.*, invisible for the radiologist during and after the procedure. It remains to be seen if the intrinsically radiopaque microspheres will perform at least as well *in vivo*, and whether the particles will be visible under clinical conditions in real time imaging within the operating theater.

## 4. Improving Microsphere-based Therapies by Controlled Local Drug Delivery

The above described microsphere-based treatments depend purely upon the presence of the microspheres for therapeutic effect. However, the efficiency of the treatment may be improved by controlled, local delivery of bioactive compounds from the spheres. For instance, when microspheres are applied as fillers or bulking agents, the release of factors that influence growth and differentiation of cells in the surrounding tissue may be considered. For non-permanent fillers, it may be important that, over time, the occupied space is slowly replaced by the desired tissue. An example is the use of degradable microspheres as drug-delivery vehicles in bone-filling formulations or scaffolds. The microspheres slowly release, for instance, bone-morphogenic protein 2 (BMP-2) or vascular endothelial growth factor (VEGF) to improve the formation of new bone in cases of critical size defects [157]. For permanent bulking agents, release of growth factors has not been attempted because there is a risk of uncontrolled cell proliferation.

The efficiency of embolization therapy may also benefit from local drug delivery. Microspheres applied for transarterial chemo-embolization (TACE) release anti-cancer drugs like doxorubicin or cisplatin [154,155,158,159,160]. The microspheres are accurately injected into the feeding arteries of a tumor, and block the circulation. Then, for instance, doxorubicin is released and can attack tumor cells from inside the tumor, creating a “double” treatment. However, since the blood flow around the embolizing spheres is blocked, it is difficult to predict the exact kinetics of local drug release inside the tumor. Additionally *in vivo* data on drug distribution in the tissue after release from microspheres is not available. Another example is ibuprofen loaded microspheres, which are intended for uterine artery embolization [9,10]. These microspheres are being investigated to combat inflammation, as well as the pain that is frequently experienced directly after the intervention [161]. This strategy is potentially more effective than systemic application of pain-killers, which have difficulty reaching the embolized uterine fibroid, since fresh blood is no longer reaching the UF. Therefore, the concentration of ibuprofen in the treated tumor is very low. The group of Laurent has reported on *in vitro* and *in vivo* ibuprofen release from PVA microspheres. It was demonstrated that *in vitro* release is dependent upon the experimental set-up [162]. *In vivo* application of ibuprofen-loaded microspheres in a sheep model demonstrated that ibuprofen was locally released, although reduction in pain could only be monitored indirectly [163].

Loading of microspheres can be divided into two basic strategies. The drug can be added during the synthesis of microspheres or can be soaked in after production.

When spheres are produced using solvent evaporation, the drug can be included in the polymer solution and subsequently, the microspheres can be formed normally. The encapsulation efficiency is determined by several factors, among which the solubility of the drug and viscosity of the polymer solution are the most obvious [15,28]. The high viscosity of the polymer solution determines the size of the microspheres, as well as the diffusion of the drug from the spheres. The temperature at which the spheres are formed, as well as the speed of solvent evaporation, can improve the encapsulation efficiency by ensuring a rapid formation of a hard surface layer, thus limiting drug loss to the aqueous, stabilizer phase [15,28]. When the drug is readily soluble in water, conditions during microsphere synthesis will have to be chosen carefully to avoid loss of the enclosed drug during the synthesis and washing steps.

The microspheres that are produced using solvent-evaporation, especially the microspheres composed of PLGA, have found widespread use as drug delivery vehicles; the degradation of the microspheres can be coupled to drug delivery [15]. Also, this material has been FDA approved for clinical use. There are several design parameters that are of importance in obtaining the required drug release profile. The molecular weight of the used polymer chains has been shown to greatly influence the rate and profile of drug release [164,165]. For example, the use of PLGA with higher molecular weight resulted in slower drug release, although some groups report a higher initial burst release, which may be due to uneven drug distribution in the microspheres. Low molecular weight (Mw) PLGA leads to faster drug release that is mostly determined by diffusion. With high Mw PLGA, drug release is not only dependent upon diffusion rate, but also upon the rate of degradation of the PLGA polymer chains [166]. Consequently, a more complex release profile is obtained that varies with the experimental conditions under which the drug release is determined [16,164]. Another parameter influencing drug release kinetics is microsphere size. When smaller microspheres are used for drug delivery, the lower surface to volume ratio should be taken into account [167,168,169,170]. The more surface area is present, the faster the initial release will be. But for microspheres that are larger than approximately > 30 μm, a different release profile is observed because the release in this case is not solely diffusion driven. The diffusion paths become longer in larger spheres, and as a result, diffusion of the drug to the sphere surface is slower [166,167]. Degradation of polymer chains in the microsphere will lead to increased diffusion rates and therefore to acceleration of drug release. The resulting curve will often show a sigmoidal shape, with the initial burst caused by accumulation of the drug in the periphery of the microspheres [165,166]. Then after a period of slow drug release, degradation of the polyester PLGA leads to reduced polymer chain length and accelerated diffusion and drug release.

When microspheres are synthesized by suspension, emulsion, or dispersion polymerization, the drug can be added to the monomer mixture before the reaction or soaked in after production. Addition of drug to the monomer mixture is, in practice, not used because it will have to survive elevated reaction temperatures, free radicals, and the organic solvents that are encountered during microsphere synthesis. Many drugs will not survive such a regime. Additionally, the drug may become entrapped inside non-degradable microspheres and will not be released, since the polymer network is too tight. All in all, the direct loading of microspheres with drug in suspension polymerization is very rarely used [171]. Instead, the drug is often soaked into the spheres after production. For this, microspheres are incubated in a concentrated drug solution. The loading efficiency is dependent upon the microsphere size (surface to volume ratio), polymer molecular weight, porosity, and time of the soaking procedure. The disadvantage of the soaking technique is that the drug seems to end up exclusively in the outer shell of the microspheres; consequently, drug release is relatively fast [16,17].

For all these drug-loaded microspheres, there are additional parameters that determine drug release kinetics. The exact application and injection site, sub-dermal or intra-vascular, will also determine, for instance, the size of the microspheres that is employed. Moreover, the stability and solubility of the drug will have to be taken into account, since most *in vitro* experiments are performed in large liquid volumes within sterile conditions without cells, proteins, and enzymes present that may influence stability and degradation.

In conclusion, the clinical use of (degradable) polymeric, drug-loaded, microspheres is complex because the controlled release *in vivo* is difficult to exactly control. However, such microspheres pose a range of opportunities to achieve new, improved, or more efficient microsphere-based treatment protocols.

## 5. Concluding Remarks and Outlook

Synthetic polymeric microspheres are frequently used in clinical practice, and the number of applications is steadily increasing. They serve diverse roles that range from bulking agents to drug delivery depots. It has become clear that microspheres have advantages that make them well suited for clinical application. First of all, microspheres are easy and relatively cheap to produce. The polymers that are currently being used for synthesis of commercial spheres are proven to be biocompatible and safe. But there are some shortcomings to the current synthetic microspheres that are rooted in the choice of the used polymers. For instance, microspheres as fillers and bulking agents tend to migrate away from the injection site. This has far-reaching consequences, among which loss of the therapeutic effect is the most obvious. It means that the patient has to undergo a series of treatments that may be advantageous for the microsphere selling companies, but which pose potential risks for the patient. Another potentially dangerous effect of microsphere migration is embolism, which can eventually lead to organ damage. However, large-scale, well controlled studies on the migration of microspheres in bulking agents and fillers are absent. An obvious improvement of microspheres would be the attachment of molecules that enhance the anchoring of the spheres within the surrounding soft tissue. The passive nature of the current spheres is clearly not sufficient to induce a proper response from the surrounding cells. Modification of the surface with bioactive molecules, for instance, with components of the extra-cellular matrix, would induce adhesion of cells, and consequently, stronger anchoring of the microspheres at the site of injection in the soft tissue.

In the case of embolization therapies, the microspheres are rather simple, and the most important development over the past decade is that drug-loaded microspheres can be employed as embolizing particles. This is most obvious in TACE, which combines the blocking of a feeding artery of a tumor with local delivery of drugs to get a maximal necrotic effect upon targeted tumor cells. Again here, mis-localization of injected microspheres is a serious problem. The major problem is that the currently commercially available microspores are not visible with the imaging techniques that are routinely used in the operating theater, *i.e.*, X-ray. The development of intrinsically radiopaque microspheres could help solve this problem. The microspheres can be observed in real time during the procedure, and when spheres reflux, *i.e.*, flow into a wrong artery, the procedure can be stopped. Then differently sized microspheres can be chosen to avoid non-targeted embolization. This feature of X-ray visibility is also suited to improving the microspheres that are used as fillers or bulking agents. In such treatments, the microspheres can also be imaged during the injection and the treatment can be adapted when necessary. The result after injection can be clearly visualized and the development of the injected material over time can be monitored. It seems unlikely that individual microspheres that migrate away from the injection site can be easily detected; this will depend upon the size of the spheres used and the quality of the imaging equipment. A loss of X-ray contrast at the site of injection will be a clear indication of microsphere migration.

Another important development will be in drug-loading strategies. The use of new, degradable polymers that have predictable degradation kinetics *in vivo* will be of great use for controlled drug delivery, especially of large bioactive molecules. Small sized drugs are easily incorporated in microspheres, although the loading efficiency is still variable. Large sized bioactive molecules are difficult to soak into microspheres, and when incorporated into a microsphere, have difficulty diffusing out. Therefore, controlled degradation of microspheres, preferably by surface erosion, instead of the bulk erosion of PLGA spheres, will be required to deliver such drugs in a controlled and orderly fashion.

Furthermore, the combination of several drugs in one microsphere, injection of several different kinds of microspheres, or the synthesis of microspheres with different layers containing different drugs, can lead to a more personalized drug delivery regime. Such sophisticated microsphere-based strategies can then be applied to obtain the most optimal drug release profile for the patient, and thus, the best possible therapy.

Finally, it is clear that microspheres are nowadays an important part of the physician’s toolkit, making the treatment of patients easier and safer. However, there are still important features that need to be added to the microspheres’ pallet to optimize their clinical use.
